# Simplified EEG/aEEG to monitor the injured brain after cardiac arrest

**DOI:** 10.1186/cc10892

**Published:** 2012-03-20

**Authors:** H Friberg, M Rundgren, E Westhall, N Nielsen, T Cronberg

**Affiliations:** 1 Lund University, Lund, Sweden; 2 Helsingborg Hospital, Helsingborg, Sweden

## Introduction

Once hemodynamics is stabilized, the main concern in the comatose cardiac arrest patient is the status of the brain and the potential recovery of brain functions. Approximately 30% of comatose cardiac arrest patients develop electrographic seizures, many of whom have associated clinical seizures that may be concealed by sedation and paralyzers. As part of the Lund coma project, we have continuously monitored and evaluated simplified EEG/aEEG in consecutive hypothermia-treated cardiac arrest patients.

## Methods

Needle electrodes corresponding to the F3 to P3 and F4 to P4 leads were applied at admission to the ICU. The Nervus NicoletOne® monitor (CareFusion Inc.) was used to display the continuous raw EEG curves as well as the amplitude integrated EEG (aEEG). The EEG data were available to the treating intensivist and were linked to the Department of Neurophysiology, where the accumulated data were interpreted once daily, 5 days a week.

## Results

Monitoring of aEEG was successfully applied in all patients. Four dominating patterns were defined; flat, continuous, suppression-burst (SB) and electrographic status epilepticus (ESE) [1]. We identified three groups of patients: one group with mild brain injury and a good outcome, characterized by a return of a continuous EEG pattern during the first 24 hours. A second group with severe brain injury and a poor outcome had a flat EEG or a SB pattern during the first 24 hours, which evolved into alfa-coma or a treatment refractory ESE. In this group, early myoclonus was common. The third group with a presumed intermediate brain injury often developed a late ESE during rewarming, from a continuous and sometimes reactive background EEG. In this third group, which presented with low brain damage biomarkers and unremarkable MR brain imaging, there were survivors, some of whom received prolonged care in the ICU [2].

## Conclusion

Simplified EEG/aEEG is easily applied and well adapted to the ICU environment. In combination with the raw EEG, the aEEG serves as a trend monitor of the injured brain in the comatose patient after cardiac arrest. The simplified EEG/aEEG helps detect ESE and is of importance for guiding anticonvulsive treatment. The evolution of the EEG pattern mirrors the natural recovery of cortical function after cardiac arrest and gives useful positive as well as negative prognostic information. Simplified EEG/aEEG serves the needs of the intensivist and has the potential to become part of a standard monitoring regimen.

## References

[B1] Rundgren M Crit Care Med 2010 38 1838 10.1097/CCM.0b013e3181eaa1e7 20562694

[B2] Cronberg T Neurology 2011 77 623 10.1212/WNL.0b013e31822a276d 21775743

